# Comparison of the Digestibility of the Major Peanut Allergens in Thermally Processed Peanuts and in Pure Form

**DOI:** 10.3390/foods3020290

**Published:** 2014-05-07

**Authors:** Soheila J. Maleki, David A. Schmitt, Maria Galeano, Barry K. Hurlburt

**Affiliations:** Southern Regional Research Center, Agricultural Research Service, U.S. Department of Agriculture, 1100 Robert E. Lee Blvd, New Orleans, LA 70124, USA; E-Mails: soheila.maleki@ars.usda.gov (S.J.M.); daschmitt2004@hotmail.com (D.A.S.); mgalea-athens@hotmail.com (M.G.)

**Keywords:** peanut, allergy, allergen, processing, digestion

## Abstract

It has been suggested that the boiling or frying of peanuts leads to less allergenic products than roasting. Here, we have compared the digestibility of the major peanut allergens in the context of peanuts subjected to boiling, frying or roasting and in purified form. The soluble peanut extracts and the purified allergens were digested with either trypsin or pepsin and analyzed by gel electrophoresis and western blot. T-cell proliferation was measured for the purified allergens. In most cases, boiled and raw peanut proteins were similarly digestible, but the Ara h 1 protein in the boiled extracts was more resistant to digestion. Most proteins from fried and roasted peanuts were more resistant to digestion than in raw and boiled samples, and more IgE binding fragments survived digestion. High-molecular-weight fragments of Ara h1 were resistant to digestion in fried and roasted samples. Ara h 1 and Ara h 2 purified from roasted peanuts were the most resistant to digestion, but differed in their ability to stimulate T-cells. The differences in digestibility and IgE binding properties of the major allergens in roasted, fried and boiled peanuts may not explain the difference between the prevalence of peanut allergy in different countries that consume peanut following these varied processing methods.

## 1. Introduction

Approximately 5% of adults and 8% of children have a food allergy, and there is evidence for an increasing prevalence. An older study indicated that peanut allergies cause the majority of the annual emergency room admissions due to food allergies and approximately 63%–67% of deaths due to anaphylaxis [1]. Most recent studies have shown an 18% increase in food allergy and a prevalence of 1.3% peanut sensitive individuals [2,3]. Despite the focus on this issue, there is still no treatment for food allergies, and the only available option is avoidance. Even with avoidance, 55% of peanut allergic individuals have at least 1–2 accidental peanut ingestions every five years [4]. With the wide number of applications for peanut and peanut products in processed foods, particularly in candy and confectionary products, and the potential for cross-contamination of peanut-free products with traces of peanuts, avoidance can be very difficult for allergic consumers. Therefore, peanut allergy is not only an increasing public health problem, but it also poses a challenge to the food industry and regulatory agencies in terms of food safety.

In order to understand the immune system-allergen recognition and response, it is important to understand the cause(s) of allergenicity in the allergenic foods and the allergenic components at a molecular level. The effects of processing on clinical symptoms caused by a food has become increasingly important, due to recent studies that demonstrate that individuals can safely become desensitized to a food by consuming that food in one processed form *versus* another [5,6,7,8,9,10]. If the processing-induced-specific changes in an allergic protein can be determined and correlated with clinical reactivity, designing safe preventative or immunotherapeutic treatments through food processing has much potential. Previously, we addressed the effects of thermal processing on some of the allergenic properties of peanut proteins [11,12]. Thermal processing, such as roasting, curing and various types of cooking, can cause multiple non-enzymatic, biochemical reactions to occur in food [13]. One of the predominant reactions that occurs during the thermal processing of foods is known as the Maillard reaction, which is important in the development of flavor and color [13]. In addition to protein cross-linking, it is known that advanced Maillard reaction products [14,15], also known as advanced glycation end-products (AGEs), could lead to the modification of amino acids, such as lysine and cysteine [16]. The majority of alterations to protein structure are due to the heat-induced interactions of sugar components with amino acids to form compounds, such as carboxymethyllysine, melanoidin and other non-cross-linking modifications to proteins that may have detrimental nutritional, physiological and toxicological consequences [13,16]. Other studies have addressed the role of food processing on the allergenic properties of ingested foods [11,12,17,18,19,20,21,22,23,24]. Some of the roasting-induced biophysical mechanisms for enhanced allergenic properties of the major peanut allergens were previously explored in a simulated roasting model [11]. Both Ara h 1 and Ara h 2 bound higher levels of IgE, and the increase in IgE binding was correlated with increased carboxymethyllysine (CML) modifications on the surface of the protein [25]. Ara h 1 was found to be inter-molecularly cross-linked to form highly stable trimers, and Ara h 2 was thought to form intra-molecular cross-links due to roasting, without forming higher orders structures. Since resistance to digestion is a classic characteristic of food allergens, we wanted to determine if different thermal processes induced different modifications of the peanut allergens, altering their stability against digestive enzymes within the context of other peanut proteins or if purified from thermally processed peanuts. The effects of different thermal processes on Ara h 1 and Ara h 2 were assessed for digestibility with trypsin and pepsin, IgE binding and stimulation of T-cells from peanut allergic individuals.

## 2. Experimental Section

### 2.1. Patient T-Cells and Sera

Sera and lymphocytes were obtained from the blood of peanut allergic individuals, which were collected after informed consent at Tulane Health Science Center (New Orleans, LA, USA) in accordance with the rules and regulations of the institutional review board. A pool of sera from previously well-characterized and -described peanut allergic patients’ sera was used in this study [17].

### 2.2. Extract Preparation and Protein Purification

Florunner peanuts were used either raw, roasted, boiled or fried, as previously described [19]. The samples were solubilized by adding 50 mg of a defatted peanut meal to 1.8 mL of a buffer containing 60 mM Tris, 1 mM EDTA and 200 mM NaCl at pH 8.5 followed by sonication and centrifugation at 5500× *g* for 15 min to remove insoluble material yielding CPE (crude peanut extract). Ara h 1 and Ara h 2 were purified as described [23,26].

### 2.3. Digestion Reactions

Trypsin digestions were set up according to Maleki *et al.* [23]. Raw, roasted, boiled or fried peanut extracts (each at a concentration of 5 mg/mL) and the purified Ara h 1 (1 mg/mL) and Ara h 2 (1 mg/mL) from raw and roasted peanuts were incubated in the presence of 1 µM trypsin (final concentration of trypsin) in PBS at pH 8.5 for various times at 37 °C. Aliquots were taken for SDS-PAGE analysis at the times indicated in each figure. Pepsin was used to make simulated gastric fluid (SGF). The peanut samples were incubated in the presence of SGF (0.5 μg/mL pepsin in phosphate buffered saline (PBS) at pH 2 at 37 °C), and aliquots were taken at the indicated time points in each figure. The digestion reaction in each time point aliquot was quenched by the addition of SDS sample buffer. Samples were then subjected to SDS-PAGE and either stained or transferred to nitrocellulose for western blot analysis. The 0 time point was taken immediately after mixing the sample with the enzymes and not before adding enzyme, so some degree of digestion may be observed.

### 2.4. SDS-PAGE and Western Blot Analysis

The samples from each time point of digestion were subjected to SDS-PAGE on 4%–20% Novex Tris-HCl pre-cast gels (Life Technologies, Carlsbad, CA, USA), where individual proteins were separated according to size and either stained with Gel-Code Blue (Pierce, Rockford, IL, USA) for 1 h, and digitally recorded, or transferred to PVDF membranes. The membrane was then blocked for 1 h using 5% Blotto (5% dry milk dissolved into PBS containing 0.5% Tween (PBST)). After blocking the membrane, the primary antibody was diluted in 5% Blotto, added to the membrane and incubated for 1 h. The custom-made antibodies used were chicken anti-Ara h 1 and anti-Ara h 2 (Sigma Immunosys, The Woodlands, TX, USA) at 1:5000. For IgE western blots, membranes were blocked in 2% Blotto for 15 min and incubated overnight with 1:10 dilution in PBST of patient sera from allergic individuals. After the incubation with primary antibodies, the membranes were washed 3 times with PBST and incubated with the either anti-chicken IgY at 1:100,000 or 1:10,000 anti-human IgE horseradish peroxidase (HRP)-labeled secondary antibody (Sigma Chemical Company, St. Louis, MO, USA) in 2% Blotto for 30 min. The membrane was then washed 3 times with PBST and incubated with ECL-Plus Western substrate (Amersham Bioscience Corp., Piscataway, NJ, USA). The signal was then visualized using a CCD camera system (Fuji Photo Film Co., Ltd., Duluth, GA, USA).

### 2.5. T-Cell Proliferation

The peripheral blood lymphocytes (PBLs) of 5 peanut allergic individuals were isolated from whole blood using standard ficoll gradient centrifugation (Sigma-Aldrich, St. Louis, MO, USA). Cells were washed and suspended in media at a concentration of 4 × 10^6^ cells/mL. For the T-cell proliferation assays, triplicate wells of a 96 well plate at 2 × 10^5^ PBLs/well were stimulated with media (control), CPE (50 μg/mL, data not shown), raw and light roasted Ara h 2 (10 μg/mL) and Ara h 1 (25 µg/mL) at 37 °C for 6 days. On Day 6, the cells were treated with [3H]-thymidine (1 µCi/well) and re-incubated at 37 °C for 6–8 h before harvesting onto glass fiber filters (Packard, Meriden, CT, USA). T-cell proliferation was estimated by quantifying the [3H]-thymidine incorporation into the DNA of proliferating cells. [3H]-thymidine incorporation is reported as the stimulation index (SI), which is defined as fold stimulation above media treated (control) cells.

## 3. Results and Discussion

**Figure 1:**
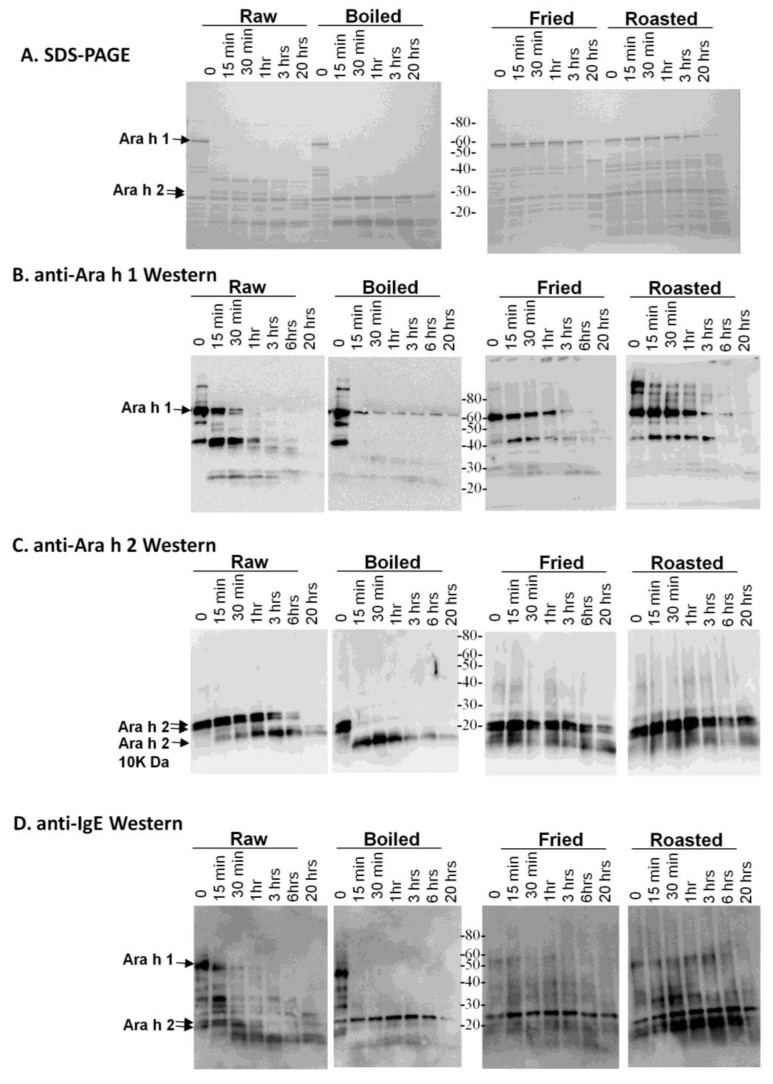
SDS-PAGE and western analysis of the digestion of raw, boiled, fried and roasted peanut extracts with trypsin. Soluble protein extracts from raw, boiled, fried and roasted peanuts were digested with trypsin and subjected to SDS-PAGE (**A**), western blot analysis with anti-Ara h 1 (**B**), western blot analysis with anti-Ara h 2 antibody (**C**) and western blot analysis with pooled human IgE sera from peanut allergic individuals (**D**).

One characteristic believed to contribute to a food protein’s allergenicity is resistance to digestion. Major peanut allergens, Ara h 1 and, especially, Ara h 2, are known to be resistant to degradation by digestive enzymes. In order to determine if diverse processing methods can alter the digestibility of thermally processed peanut proteins, soluble extracts were made from raw, roasted, boiled and fried peanut extracts, which were then subjected to digestion with trypsin (Figure 1) several times prior to SDS-PAGE analysis (Figure 1A).

This figure demonstrates that the higher molecular weight protein bands, such as Ara h 1, are more resistant to digestion in the fried and roasted samples than in the raw and boiled samples. The digestion pattern is also different between raw and boiled samples, in which two main lower molecular weight bands, which could be fragments of other proteins, persist after 20 h of digestion with trypsin. An anti-Ara h 1 western blot on the same extracts shows that the Ara h 1 in the boiled sample is more resistant to digestion than in the raw sample (Figure 1B). Higher order structures or oligomers of Ara h 1, previously shown to exist in the simulated roasting model, are clearly recognized by the anti-Ara h 1 antibody in this western blot. Ara h 1 is more resistant to trypsin digestion in all of the thermally processed peanuts compared to the raw peanut. Digestion of Ara h 1 with trypsin *in silico* yields 84 fragments, with the largest being 3.8 kDa. Therefore, if complete digestion occurred, very few bands in the ~3–4 kDa range would be visible on the percentage of SDS-PAGE used here. It is highly likely that many trypsin digestion sites are blocked by the protein structure and or by thermal processing-induced chemical modifications An anti-Ara h 2 western blot demonstrates that the Ara h 2 in raw peanut is more stable than the boiled sample, but Ara h 2 in roasted and fried samples are more resistant to trypsin digestion than in both the raw and boiled peanuts (Figure 1C). The known Ara h 2 10 kDa digestion-resistant band [27] can be seen below the intact Ara h 2 doublet in all of the extracts. Digestion of Ara h 2 with trypsin *in silico* generates 21 fragments, the largest of which is 2 kDa. These same samples were assessed for IgE binding using western blot analysis to determine the effect of the processing on the IgE recognition pattern of allergens within the context of the extracts (Figure 1D). It appears as though the IgE binding proteins in the boiled peanut, particularly the higher molecular weight ones, are the most significantly reduced and rapidly digested with trypsin.

**Figure 2:**
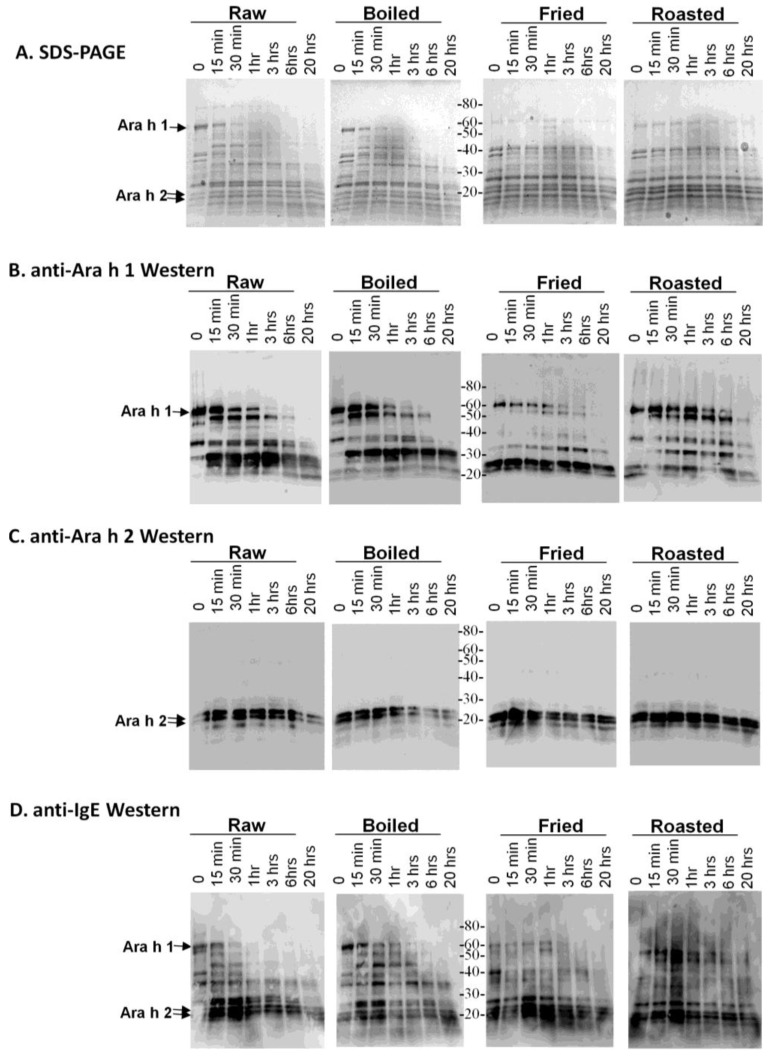
SDS-PAGE and western analysis of the digestion of raw, boiled, fried and roasted peanut extracts with pepsin. Soluble protein extracts from raw, boiled, fried and roasted peanuts were digested with pepsin and subjected to SDS-PAGE (**A**), western blot analysis with anti-Ara h 1 antibody (**B**), western blot analysis with anti-Ara h 2 antibody (**C**) and western blot analysis pooled human IgE sera from peanut allergic individuals (**D**).

The raw, boiled, fried and roasted peanut extracts were subjected to digestion with pepsin (Figure 2). The SDS-PAGE indicates that most of the peanut proteins are more resistant to digestion with pepsin than to trypsin and that the proteins in the fried and roasted peanut samples seem to be minimally altered due to pepsin treatment over a 20-h period (Figure 2A). Digestion of Ara h 1 with pepsin *in silico* yields 33 fragments, the largest of which is 5 kDa. An anti-Ara h 1 western blot of the pepsin digests indicates that following 20 h of digestion, a large Ara h 1 digestion fragment, immediately below the intact Ara h 1 and several smaller Ara h 1 fragments of <40 kDa survive digestion in the roasted sample. Digestion of purified Ara h 1 with pepsin has also been reported to result in relatively large fragments capable of binding IgE [28]. The digestion pattern of Ara h 1 in the raw, boiled and fried extracts are similar, but the fragments that survive after 20 h are different in the fried peanut extracts. Chemical modification of digestion sites and surrounding amino acid residues can alter the digestion patterns by an enzyme, often by blocking the sites to be cleaved. These results indicate that the chemical or structural modifications to Ara h 1 in boiling and frying are similar in some aspects, but can also be vastly different.

**Figure 3:**
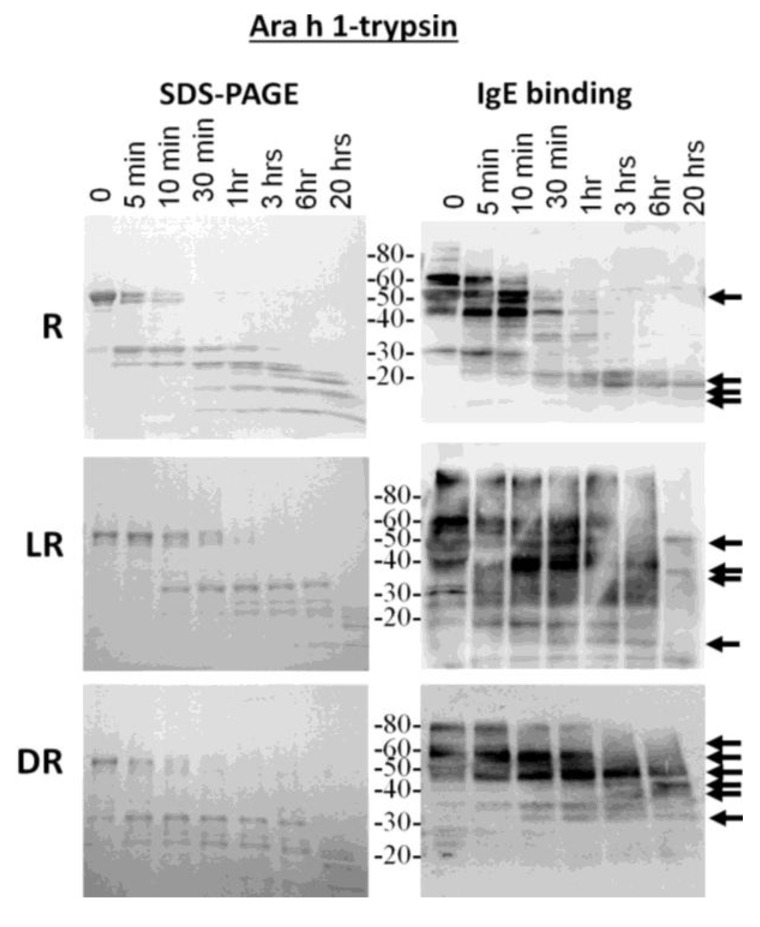
SDS-PAGE and IgE western analysis of the digestion of raw (R), light roast (LR) and dark roast (DR) Ara h 1 with trypsin. Raw Ara h 1 (*top*), light roast Ara h 1 (*middle*) and dark roast Ara h 1 (*bottom*) were digested with trypsin for the designated amounts of time. Sample taken prior to addition of trypsin is Time 0. The molecular weights of the marker proteins are indicated on the right.

Because the most diverse digestion patterns were seen between raw and roasted peanut extracts, the major allergens, Ara h 1 and Ara h 2, were purified from raw (R), light roast (LR) and dark roast (DR) peanut and compared for their digestibility with trypsin and pepsin (data not shown for pepsin digestion). R, LR and DR Ara h 1 were subjected to trypsin digestion for the indicated times and resolved by SDS-PAGE (Figure 3, left). The IgE binding ability to the digested fragments was assessed by western blot (Figure 3, right). The R Ara h 1 is completely digested into smaller fragments following 30 min of incubation in the presence of trypsin. A strong 35-kDa band appears and survives digestion for approximately 1 h, as the intact Ara h 1 band is digested. Fragments smaller than 35 kDa survive digestion overnight, two of which are recognized by IgE, similar to what is seen in the case of the Ara h 1 digested within the context of raw peanut proteins; whereas the intact LR Ara h 1 can be seen after 3 h and DR intact Ara h 1 after overnight digestion with trypsin. The same 35-kDa fragment seen in R Arah 1 appears and survives more than 3 h in the presence of trypsin in both the LR and DR samples. While there are some higher molecular weight (>30 kDa) proteins present following 1 h of digestion in the R sample, after 20 h, some fragments of Ara h 1, approximately 25 kDa and below, remain undigested. When Ara h 1 digestion was followed within the context of raw peanut proteins, these bands are not seen after 20 h (Figure 1B). The molecular weights of the four fragments visible following overnight digestion with trypsin in SDS-PAGE, in all three digestion reactions, are ~22, 18, 13 and 13 kDa, three of which are recognized by IgE. This indicates that even though the resistance to digestion increases with the degree of roasting, the predominant trypsin digestion sites remain the same. However, the bands that are recognized by serum IgE are significantly different. The IgE binding in the DR sample is only to the higher molecular weight bands and the smaller bands in the raw extracts following 20 h of digestion. This indicates that the fragments of allergens that are recognized by serum IgE of allergic individuals change with roasting. The Ara h 1 seems to be less detectable or more digestible within the context of peanut proteins. We attribute this to both the presence of more Ara h 1 to digest and visualize in the pure samples; however, the fragments that survive digestibility within the context of R and LR peanut are similar to the purified proteins.

**Figure 4:**
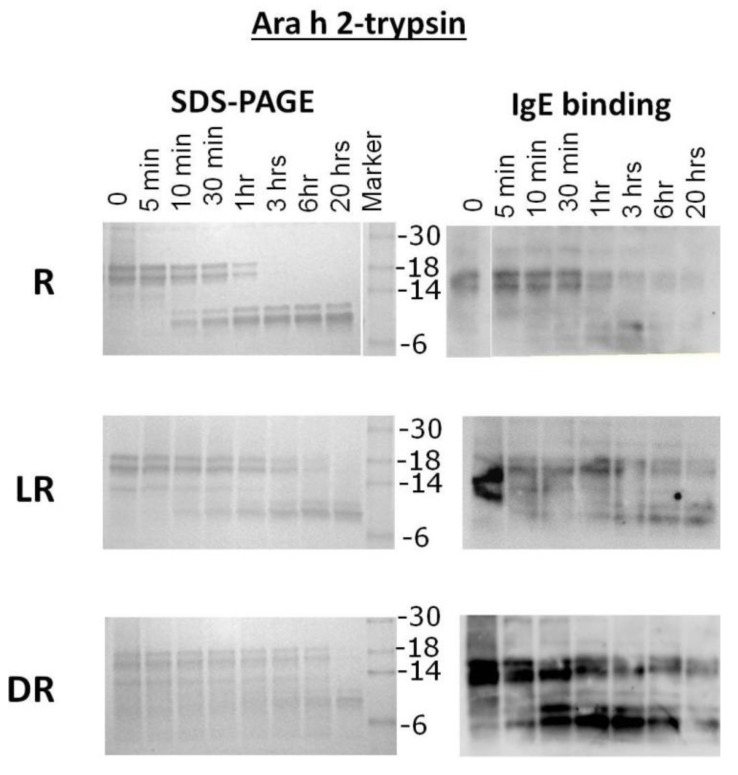
SDS-PAGE analysis and IgE western analysis of the digestion of raw, light roast (LR) and dark roast (DR) Ara h 2 with trypsin. Raw Ara h 2 (R), light roast Ara h 2 (LR) and dark roast Ara h 2 (DR) were digested by trypsin for the designated amounts of time of 5 min (5’) to overnight (O/N). Other lanes are labeled Ara h 1 and Ara h 2 control (C), undigested (U) and molecular weight marker. The molecular weights of the marker proteins are indicated on the right.

Purified samples of R, LR and DR Ara h 2 were subjected to trypsin digestion for the indicated times and resolved by SDS-PAGE (Figure 4). The intact R Ara h 2 was completely digested into smaller fragments after 1 h of incubation in the presence of trypsin. Furthermore, two fragments, a 10- and a 12-kDa band, appear as the intact Ara h 2 band disappears. These trypsin-resistant bands survive even after overnight digestion. The 10 kDa fragment becomes stronger over time, while the 12-kDa band maintains the same intensity after 30 min. In the IgE binding assay, the intact LR and DR Ara h 2 and smears thereof can both be seen after overnight digestion with trypsin. This indicates that the LR Ara h 2 and DR Ara h 2 are both more resistant to digestion with trypsin. In both LR and DR Ara h 2 samples, the 10- and 12-kDa bands, also seen in R Ara h 2, appear following the first 5–10 min of digestion, but the 12-kDa fragment appears as a smear. Interestingly, as seen in the trypsin digestion of Ara h 1, the surviving fragments are the same in all three (R, LR and DR) digestion reactions. This finding indicates that the trypsin digestion sites are not altered due to a higher degree of roasting. Interestingly, even though in SDS-PAGE, the intensity of the intact Ara h 2 is significantly decreased over time, the IgE binding remains similar, which indicates that more IgE is binding to the roasted samples. Both Ara h 1 and Ara h 2 from roasted peanuts and fragments thereof are more resistant to digestion with trypsin due to increased chemical blocking or unknown alterations of the existing digestion sites with increased time of roasting. Purified Ara h 2 digestion did not change significantly from digestion within the context of peanut proteins, which is not surprising, as the digestion sites are not altered.

**Figure 5:**
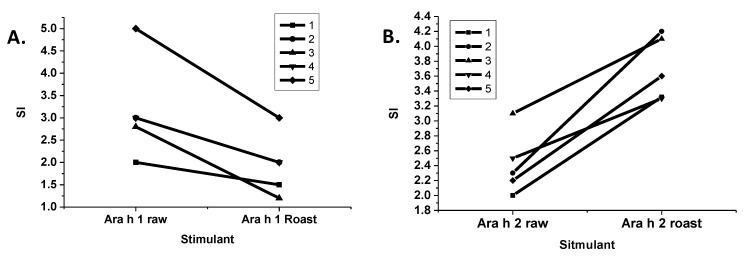
T-cell response to Ara h 1 and 2 purified from raw and roasted peanut extracts. Lymphocytes from five peanut allergic individuals were stimulated with Ara h 1 (**A**) or Ara h 2 (**B**) purified from either raw or roasted peanuts. The stimulation index (SI) is shown on the *y*-axis.

The stimulation of T-cells by purified R and LR Ara h 1 and Ara h 2 were compared (Figure 5). Interestingly, T-cell stimulation by LR Ara h 1 was significantly reduced in comparison to R Ara h 1, and the opposite was true for Ara h 2. It is known that if the IgE binding sites of an allergen are eliminated, while maintaining the T-cell proliferative characteristics of that allergen, then it can be utilized as an effective immunotherapeutic tool that alters a T-helper 2 (Th2), or an inflammatory response to a Th1 or a tolerant response [29]. In this case, the Ara h 1 allergen has a higher IgE binding and is more resistant to digestion, but T-cell proliferation is reduced. On the other hand, roasted Ara h 2 is more resistant to digestion, binds higher IgE and causes higher T-cell stimulation following roasting, indicating that Ara h 2 is more immunogenic. This is consistent with findings in the field that Ara h 2 is the most potent allergen in peanut [30].

The effects of roasting on IgE binding and the allergenic potency of peanut allergens has been under debate for a long time. One study showed that in a simulated roasting model of heating crude peanut extract (CPE), Ara h 1 and Ara h 2 in the presence of reducing sugars (55 °C for 10 days), in solution, enhanced IgE binding [11]. In another similar study, the CPE and purified Ara h 1 and Ara h 2 were dried in the presence and absence of glucose (+g and −g, respectively) prior to heating at 145 °C for 20 min [31]. Following solubilization, they found that IgE binding to Ara h 1 +g was significantly reduced, but the capacity of mediator release increased. Meanwhile, both IgE binding and mediator release with Ara h 2 +g and −g was reduced. Ara h 1 purified from roasted peanuts was shown to bind higher levels of IgE than raw peanuts [17]. This observation was attributed to chemical modifications rather than major structural alterations, and the specific Maillard reaction products on the roasted Ara h 1 were identified [17,24]. Furthermore, AGE modifications were found on Ara h 1 and Ara h 3 in both raw and roasted peanut extract, but not on Ara h 2. The receptor for advanced glycation end products (RAGE) binds selectively to Ara h 1 derived from peanut extract, whereas the analysis failed to demonstrate Ara h 2 binding to RAGE. Recombinant Ara h 1 with no AGE modifications did not bind RAGE; however, after AGE modification with xylose, recombinant Ara h 1 bound to RAGE. Perhaps, the reduced AGE modifications of Ara h 2 allow more potent IgE and T-cell epitope exposure. The ability of Ara h 2 to be processed better by antigen presenting cells, due to reduced glycation, can explain the enhanced T-cell proliferation compared to the reduction seen with roasted Ara h 1. In another study, a combination of purified Ara h 2/6 from raw peanuts was heated in solution (110 °C for 15 min) and +g and −g [32]. Roasted Ara h 2/6 was also purified for comparison. They found no differences in T-cell proliferation with the raw, heat-treated and roasted Ara h 2/6, but the raw heated sample bound less IgE, due to denaturation, hydrolysis and aggregation. Discrepancies such as these can be attributed to different methods of experimentation, such as protein extraction, purification, glycation, temperatures of heating, *etc.* For example, we have found that if purified proteins are heated in the presence or absence of sugar at high temperatures for even short periods of time, then it is as if the protein or the food extract has been charred, and the subsequent findings may not apply to the actual ingested form (unpublished observation). This observation is consistent with the findings in the previous study with Ara h 2/6 that showed a significant difference between the structure and immunological properties of a purified protein glycated rapidly at high temperature and the protein purified from roasted peanuts. In the present study, we chose to assess Ara h 1 and Ara h 2 within the context of peanuts and purified from roasted peanuts in order to study the actual digestion and immunological response of these proteins in the ingested form, as opposed to using model systems.

## 4. Conclusions

Our results show that, while model systems are highly effective in understanding molecular events in foods, it is important to understand the effects of food processing on allergenicity and on the individual allergens in order to develop effective tools for research, diagnosis, detection and immunotherapy. While it has been shown that different processes influence the allergenic properties or immunogenicity of certain foods, it has not been shown that processing can influence sensitization or the original development of allergy to a particular food. Animal models or human studies on the ability of differently processed foods to sensitize or tolerize could be useful in assessing the influence of processing on epidemiology and the development of food allergy in various countries.
